# Bayesian inference of molecular kinetic parameters from astrocyte calcium imaging data

**DOI:** 10.1016/j.mex.2022.101825

**Published:** 2022-08-23

**Authors:** Ivan V. Maly, Wilma A. Hofmann

**Affiliations:** Department of Physiology and Biophysics, Jacobs School of Medicine and Biomedical Sciences, State University of New York at Buffalo, Buffalo, NY 14221, USA

**Keywords:** Schizophrenia, 22q11.2 deletion syndrome, Glia, Model-based inference, Li-Rinzel model, Calcium dynamics, Calcium signaling, VBA, MIN1PIPE

## Abstract

Model-based Bayesian inference from high-content data obtained on live specimens is a burgeoning field with demonstrated applications to neuroscience. In parallel, computer vision methods for extracting the calcium signaling information from imaging data have advanced in application to neuronal physiology. Here, we are describing in detail a method we have recently developed to study calcium dynamics in astrocytes, which combines computer vision with model-based Bayesian learning to deduce the most likely molecular kinetic parameters underlying the observed calcium activity. As reported in the companion experimental study, this method allowed us to identify the key molecular changes downstream of a multi-gene deletion modeling the human 22q11.2 deletion syndrome, the most common human microdeletion and the genetic factor with the highest penetrance for schizophrenia.

• Methodological details are laid out, from our imaging approach to our adaptation of the VBA-CaBBI algorithm previously developed primarily for brain functional imaging data.
• The analytical pipeline is suited for further applications to glial cells and adaptable to other cell types exhibiting complexcalcium dynamics.

## Graphical abstract

**Figure:**
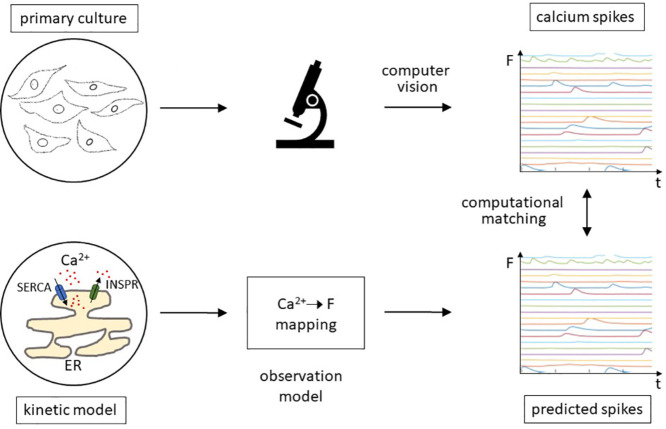
Flow diagram of experimental setup

Specifications Table

**Table:** 

**Subject area**	Neuroscience
**More specific subject area**	Computational neuroscience
**Protocol name**	Bayesian inference from intracellular calcium imaging data
**Reagents/tools**	GCaMP6 associated adenovirus vector (SignaGen; Frederick, MD) DMi8 microscope (Leica, Wetzlar, Germany) MATLAB software (Mathworks, Natick, MA) MIN1PIPE software package (Lu et al., 2018) VBA software package (Daunizeau et al., 2014) CaBBI software package (Rahmati et al., 2016)
**Experimental design**	Primary astrocytes are isolated from neonate mice, subcultured, and transduced with a fluorescent calcium indicator protein. Intracellular calcium activity is recorded with a microscope and individual temporal profiles are extracted from the video data using the MIN1PIPE algorithm. The profiles are fed into the Bayesian inference package (CaBBI-VBA) modified to utilize a calcium kinetic model suited to astrocytes.
**Trial registration**	N/A
**Ethics**	The animal procedures were approved by the University at Buffalo Institutional Animal Care and Use Committee and are consistent with the recommendations of the American Veterinary Medical Association.
**Value of the Protocol**	• Allows to dissect complex calcium imaging data in terms of the underlying molecular kinetics • Minimal human supervision once a suitable kinetic model is specified • Adaptable to study complex genetic and pathophysiological conditions

**Description of protocol:**

## Background

Studies of calcium signaling in brain cells, including astrocytes, primarily involve the expression and imaging of fluorescent calcium indicator proteins [2,^6^,17]. Our approach to analyzing the dynamics of intracellular calcium in astrocytes is based on making suitable modifications to the previously developed Bayesian method for analyzing calcium dynamics in neurons, known as CaBBI (calcium biophysical Bayesian inference, Ref. [15]). This approach is supplemented by adding a computer-vision step to automatically extract the temporal profiles of calcium indicator fluorescence in individual cells from the imaging data, using MIN1PIPE [9], an algorithm previously developed for neuronal applications.

## Method details

### Cell culture and treatment

The *Df1*/^+^ mouse line, a transgenic model of the human 22q11.2 deletion [8,14], was maintained on the C57BL/6 J genetic background as previously described [18]. *Df1*/^+^ mice were kindly provided by Dr. Stanislav S. Zakharenko, St. Jude Children's Research Hospital (Memphis, TN). Primary cultures were derived from cortices of neonate hemizygous *Df1* mice (*Df1*/^+^) and their wild-type (^+^/^+^) littermates (control) as previously described [16]. Specifically, the cortices were triturated, digested with trypsin (Thermo Fisher, Waltham, MA), and seeded in flasks (Corning, Corning, NY) coated with polylysine (Thermo Fisher).

After 1 week in culture, the cells were immunomagnetically purified using the Miltenyi Biotech (Auburn, CA) anti-ACSA2 astrocyte purification kit according to the manufacturer's protocol, as described previously [1,5]. Purified cells were maintained in ScienCell (Carlsbad, CA) complete astrocyte medium-animal. Two weeks later, the cells were transduced with adeno-associated viral vectors (AAV; multiplicity of infection of 100,000) expressing the fluorescent intracellular calcium indicator fusion protein, GCaMP6m (SignaGen; Frederick, MD) [2] in accordance with the supplier's protocol. Specifically, the vector particles were added to the cell culture medium at the specified concentration, and 2 days were allowed for expression before imaging.

For imaging, cells were grown in Labtek (Grand Rapids, MI) optical glass-bottom chambers coated with polylysine (Thermo Fisher, Waltham, MA). Prior to imaging, the medium was exchanged for Hanks balanced salt solution with calcium (1.26 mM), magnesium (0.89 mM), and d-glucose (5.6 mM; Thermo Fisher), and the culture was allowed to equilibrate for 40 min. In the experiments with thapsigargin (Thermo Fisher), the inhibitor was added to the medium at 200 pM on the microscope stage.

## Microscopy

Video recording was performed on a Leica DMi8 inverted fluorescence microscope equipped with an environmental control chamber for live-cell imaging. The light-emitting diode excitation power was set to 1% and attenuated by 85% using a neutral density filter to minimize light exposure. ROIs (from which the individual fluorescence time traces were extracted) were defined automatically by MIN1PIPE as spatially disjoint, temporally uncorrelated areas of strong fluorescence variation throughout the entire astrocyte. Images were acquired in a rapid time-lapse fashion (1 Hz) over 3 min with the excitation shutter closed between frames using Leica LAS X software and a monochrome cooled camera (Leica DFC9000GTC). A field of view was selected blindly and the culture was imaged in a predetermined geometrical pattern around the first field, using the computerized stage control. Each field of view was recorded once, and a field diaphragm was used to restrict excitation light exposure to the area being imaged.

## Image analysis

Imaging data were analyzed in MATLAB R2020b software (MathWorks, Natick, MA). The computer vision algorithm in the MIN1PIPE package (default settings, version v3 alpha; https://github.com/JinghaoLu/MIN1PIPE/releases/tag/v3.0.0) was used to automatically identify individual active regions in the image sequences and extract fluorescence time courses from them [9]. MIN1PIPE accepts raw calcium fluorescence videos, removes the background, finds separated ROIs and outputs deconvolved calcium fluorescence traces. The algorithm contains an enhancing module, which minimizes fluctuations and background unevenness, and a signal extraction module that identifies the ROIs and corresponding calcium traces without the need to specify unknown parameters a priori. MIN1PIPE extracts signals in two steps: the step known as “seeds-cleansing,” to detect the set of real ROIs, followed by a version of constrained nonnegative matrix factorization (CNMF) applied spatiotemporally to separate ROIs. Thus, first, an over-complete set of seed ROIs is generated at random, which contains all potential ROI centers and thus includes a large number of false positives. This set is coarsely culled by applying a two-component Gaussian mixture model (GMM) to the peak-valley difference in the corresponding fluorescence traces, with the components being the real and false-positive populations. The remaining false positives are removed using recurrent neural networks (RNNs) trained offline. ROIs are further culled by preserving only the ones with maximum intensity among those that are spatiotemporally close. At the second step, CNMF is applied iteratively to update the outlines of ROIs and the deconvolved temporal traces. The unique feature of the CNMF algorithm in MIN1PIPE is that it operates on the original image intensity data at each iteration of updating the outlines and traces. This is especially important in the present application to astrocytes, were information beyond the mere timing of the spikes is of first importance. It also allows the optimization to be parallelized in MIN1PIPE, which makes the run times on wide fields such as obtainable *in vitro* manageable. The structural element size for the morphological opening operation was set according to the image scale (13 μm/10 pixels).

## Kinetic inference

### Overview of the method

Kinetic parameters of the cell calcium system that are most likely, given the fluorescence time courses, were inferred using a suitably modified CaBBI module [15] for the VBA package [3], which implements a variational Bayesian approach [4]. CaBBI infers the cellular kinetics controlling the intracellular calcium concentration from fluorescent calcium indicator time series. VBA describes a specific application with a generative model that consists of evolution and observation functions: the former captures the intrinsic dynamics of the system in question and the latter quantitatively maps these onto the measurement readout from the experiment. In a specific application, the VBA functionality is called by a MATLAB script specific to the problem, which also specifies the evolution and observation functions to be used. The CaBBI module supplies this script and the functions that are specific to calcium kinetics in neural cells.

The original CaBBI operates with kinetic models for action potential-driven calcium kinetics in neurons [15]. To apply the same method to astrocytes, the kinetic models were replaced by the Li-Rinzel model for intracellular calcium kinetics [7,11] (see also, e.g., [12]). This model considers the exchange of calcium ions between cytosolic and endoplasmic pools and incorporates the kinetics of SERCA, the inositol trisphosphate receptor (InsPR), and ligand-independent calcium leak from the endoplasmic reticulum (ER). InsPR kinetics capture the intrinsic inactivation, potentiation by cytosolic calcium, and activation by inositol triphosphate (IP3). In addition, the Li-Rinzel model considers the production and degradation kinetics of IP3, the former being dependent on the cytosolic calcium concentration. We coded this model as an evolution function for use in the VBA-CaBBI environment. All parameters were held at their previously validated values (see [12]) or varied around these values as prior means in the Bayesian inversion procedure. Specifically, the parameters that could be directly affected by the expression levels of the relevant proteins (extensive parameters) were varied: the rate constants of each of the calcium fluxes listed above and of the production and degradation of IP3. Finally, to account for the properties of GCaMP6m, the value of the dissociation constant in the CaBBI observation function was set to 167 nM [2].

In sum, the Bayesian inference approach to calcium kinetics in astrocytes that is presented here is implemented by running CaBBI with a new evolution function in the MATLAB environment with the VBA toolbox installed. The new function encodes the kinetic equations described below.

### Kinetic model

Our implementation of the kinetic model for spontaneous intracellular calcium variations in astrocytes follows Nadkarni and Jung's [12,13] application of the Li-Rinzel model [7] that is used as the basic template in most models to study astrocytes [11]. Specifically, the following differential equations are solved:

(1) $$\begin{array}{ccc} & & \frac{dC_{cs}}{dt}=J_{c}-J_{s}+J_{l},\\ & & \frac{dq}{dt}=a_{2}d_{2}\frac{p+d_{1}}{p+d_{2}}\left(1-q\right)-a_{2}C_{CS}q,\\ & & \frac{dp}{dt}=-\frac{1}{\tau }\left(p-p_{0}\right)+v_{p}\frac{C_{CS}+0.2k_{p}}{C_{CS}+k_{p}}. \end{array}$$

Here, *C_CS_* is the concentration of the calcium ion in the cytosol, *q* is the InsPR recovery variable, and *p* is the concentration of IP3. The model is closed by the following conservation relationship:

(2) $$C_{ER}=\frac{1}{c_{1}}\left[C_{0}\left(1+c_{1}\right)-C_{CS}\right],$$

where *C_ER_* is the concentration of calcium in the ER, *C*_0_ is the total concentration of calcium ion in cell, and *c*_1_ is the volume ratio between the ER and the cytosol. The fluxes through InsPR (*J_c_*) and SERCA (*J_s_*) and the leak flux (*J_l_*) are defined as follows:

(3) $$\begin{array}{ccc} & & J_{c}=v_{1}{\left(\frac{p}{p+d_{1}}\right)}^{3}{\left(\frac{C_{CS}}{C_{CS}+d_{5}}\right)}^{3}q^{3}\left(C_{ER}-C_{CS}\right),\\ & & J_{s}=v_{s}\frac{C_{CS}^{2}}{C_{CS}^{2}+k_{s}^{2}},\\ & & J_{l}=v_{2}\left(C_{ER}-C_{CS}\right). \end{array}$$

The values [12,13] of the parameters in the above expressions Eqs. (1)–((3)) are given in Table 1.

**Table 1:** Model parameter values.

Parameter	Value
*v_s_*	0.9 μM/s*
*C*_0_	2 μM
*v*_2_	0.11 s^–1^*
*k_s_*	0.1 μM*
*a*_2_	0.2
*d*_1_	0.13
*d*_2_	1.049
*d*_5_	0.08234
*v*_1_	6 s^–1^*
*c*_1_	0.185
τ	7.14, s*
*p*_0_	0.16 μM
*v_p_*	0.13 μM/s*
*k_p_*	1.1

⁎ Varied in the model inversion.

### Observation model

The cytosolic calcium concentration from the intracellular calcium kinetics model (*C_CS_*, Eq. (1)) is used to predict the calcium indicator fluorescence level. The CaBBI [15] approach to this task is encapsulated in the following observation function:

(4) $$g\left(C_{CS}\right)=\kappa_{F}\frac{C_{CS}}{C_{CS}+K_{d}}+d_{F}.$$

Here, *K*_d_ is the dissociation constant of the indicator (set to 167 nM for our experimental conditions, see above), and *κ_F_* and *d_F_* are the scale and translation parameters that are determinable in the model inversion and account for the conditions specific to each observation (gain and background levels). The output of the observation function is evaluated directly against the recorded fluorescence in the model inversion. See the next section for the algorithm of determining the observation function parameters from data.

### Bayesian model inversion

In the VBA [3] approach to approximate Bayesian inversion for biological time series models, hidden states (*x*) of the system are updated at each time point, *t*, using the deterministic evolution function *f* (kinetic model, above) and an assumption of additive noise:

(5) $$x_{t+1}=f\left(x_{t},\theta \right)+\eta_{t}.$$

A similar approach is taken to the predictions of the observable, *y*, using the observation function *g*:

(6) $$y_{t}=g\left(x_{t},\varphi \right)+\varepsilon_{t}.$$

In these expressions Eqs. (5), ((6)), *θ* and *φ* are the parameters of the respective function, whereas *η* and *ε* are mean-zero Gaussian variables. Specifically, we have

(7) $$\begin{array}{c} \eta_{t}∼N\left[0,{\left(\alpha Y_{x}\right)}^{-1}\right],\\ \varepsilon_{t}∼N\left[0,{\left(\sigma Y_{x}\right)}^{-1}\right], \end{array}$$

with *Y* being the corresponding inverse covariance matrices. In our case, *y* corresponds to the recorded fluorescence, while *x* is the set of dependent variables in the cell kinetic model (*C_CS_, q, p*). Appropriate accuracy of the kinetic model integration between time points *t* must be ensured, and this is achieved with the use of the “microtime” resolution setting as detailed below. Together, the evolution and observation models Eqs. (5), ((6)) comprise the generative model, *m*.

The generative model inversion, given the data, is based on the variational Bayesian approach and Laplace approximation [3]. Given *m* and *y_t_*, we aim to infer the moments of the posterior distributions *p*(*υ*|*y_t_*, m) on *υ* = (*x_t_, φ, θ, α, σ*). The conditional mean *μ* and covariance Σ are updated iteratively by optimizing *F*(*Q, y_t_*), termed free energy, with respect to *Q*(*υ*), the approximate posterior density. The method assumes that *Q* can be represented as a product of the marginal posterior densities. The Laplace (Gaussian) approximation is used for each of these, except for the precision parameters *α* and *σ*, which are modeled with gamma distributions. So, for the underlying kinetic parameters of interest, we have

(8) $$Q\left(\theta_{i}\right)\approx N\left(\mu_{\theta_{i}},\Sigma_{\theta_{i}}\right).$$

*F*(*Q, y_t_*) constitutes the lower bound on the logarithm of the model evidence. It is computed as the difference of the log-evidence and the Kullback-Leibler divergence of the approximate from true posterior density:

(9) $$F\left(Q,y_{t}\right)=\text{ln}\;p\left(y_{t}|m\right)-D_{KL}\left[Q\left(\upsilon \right)∥p\left(\upsilon |y_{t},m\right)\right].$$

A version of the Gauss-Newton method is used for the iterative stepping, and a predefined limited number of subsequent observation time points is used to update the hidden states at the given time point instead of the full time series. Full computational details and references for the VBA method can be found in the cited work [3].

### Software versions and settings

MATLAB R2020b (MathWorks, Natick MA) was used with VBA v1.9.2 (https://github.com/MBB-team/VBA-toolbox/releases/tag/v1.9.2) and CaBBI distributed as part of the same. Default parameter settings were used. Microtime resolution was selected as the method of integration of the underlying dynamical system (the Li-Rinzel model), whereby the integration step was 0.1 s, i.e., ten times smaller than the image acquisition interval. This ensures stability of time integration in the stepping procedure that is part of VBA, taking into account the comparative stiffness of the dynamic system of intracellular calcium kinetics. In VBA, the microtime regime allows integration of the underlying dynamic system with a different (smaller) time step than the one used to approximate the data in the Bayesian inversion. Thus, the time step in the integration of the kinetic model can be selected independently of the optimal experimental data acquisition interval. In the present application, this capability is essential, since integration of stiff systems requires small time increments.

### Priors

In the VBA-CaBBI environment, the evolution parameter priors are Gaussian. The priors used are specified in Table 2. In accordance with the approach described above, the prior means of the kinetic parameters were equal to their values in the Li-Rinzel model. The other prior means and variances (Table 2) were either left at the values selected in the referenced version of CaBBI or—where the parameters such as the initial conditions were new—set to the correct order of magnitude according to the Li-Rinzel model (means) and CaBBI (analogous variances).

**Table 2:** Priors.

Parameter	Mean	Variance (on the exponent, where noted)
[Ca^2+^]*_t_* _=_ _0_, cytosolic	0.1 μM	0.05 μM^2^
InsPR recovery variable at *t* = 0	0.5	0.2
[IP3]*_t_* _=_ _0_	0.5 μM	0.2 μM^2^
SERCA *V*_max_ (v_s_)	0.9 μM/s	2 (exp)
InsPR *k* (*v*_1_)	6 s^–1^	2 (exp)
ER leak *k* (*v*_2_)	0.11 s^–1^	2 (exp)
IP3 τ (lifetime)	7.14 s	2 (exp)
IP3 synthesis *k* (*v_p_*)	0.13 μM/s	2 (exp)
SERCA *K*_m_ (*k_s_*)	0.1 μM	2 (exp)

Non-negativity of the variable parameters was ensured through the transformation approach, as used in VBA and CaBBI. Specifically, the physical parameter was represented as a product of a constant equal to the parameter's physical prior mean and an exponential factor whose exponent was the variable parameter in the computational inversion. Where this is the case, the logarithmic transformation is noted in Table 2. The dimensional prior mean value given in the table in these instances is the physical prior mean, whereas the corresponding computational inversion parameter had a zero prior mean and the dimensionless prior variance given in the table.

The use of the exponential transformation results in a different form of distribution in the parameter space compared with what might be achieved with non-transformed parameters. Any influence of this transformation on the efficiency of optimization may be of interest in future development of the application of the Bayesian method to intracellular calcium. In general, the nonlinearity of the underlying kinetics results in very complex distributions in the parameter space, as can be seen in the cited prior work that used a different (non-variational) optimization method [19]. Any effect of the additional skew introduced by the exponential transformation will have to be viewed on this background, which makes it unlikely to be a controlling factor.

### Convergence criteria

Individual GCaMP6m fluorescence traces constituted the datasets for independently run VBA inversions (one inversion per trace), resulting in inference of parameter sets characterizing the individual active regions. Each inversion was run for a maximum of 100 iterations using the default minimum increment on the free energy function (0.02). Runs returning a positive state noise precision hyperparameter were accepted. While not censored by fit of the data in the classical sense, it was informally verified that such runs resulted in an adequate fit (*R*^2^ > 90%). Informal analysis also showed that there was wide separation of the so-defined successful from unsuccessful runs in any dimension commonly used to evaluate data fit, and that the unsuccessful runs could be attributed to the comparative stiffness of the Li-Rinzel model and the relatively rapid stepping scheme selected for reasons of overall computational efficiency. The data (fluorescence traces) causing the rejected inversion runs were excluded from the analysis. In each experimental group, it was formally verified that the rejection rate did not exceed 5% (usually amounting to 0–3%).

### Descriptive statistics and group comparisons

Posterior mean values of parameters estimated from fluorescence traces were treated in the frequentist framework as parameter sets characterizing the corresponding individual regions. This approach, employed in a Bayesian single-cell study [19], emulates standard handling of direct experimental cell measurements. Summary statistics were compiled across the active regions from the same experimental group. Group means and standard deviations calculated in this manner are presented for descriptive purposes (Table 3). Statistical significance of group differences was evaluated using the Kolmogorov-Smirnov test on the distributions of the given parameter among the active ROIs of control and *Df1*/+ astrocytes. This allowed us to identify SERCA *V*_max_ and ER leak *k* as the parameters that were significantly altered in the *Df1/+* astrocytes, as a first application [10] of the described method. Experiments with the SERCA inhibitor thapsigargin, which affects both parameters due to the reverse flux through SERCA being one of the mechanisms of ER leak, were conducted [10] to confirm the power of the method to selectively detect changes in these parameters (Table 3).

**Table 3:** Summary statistics of individual posterior means.

Parameter	Untreated wild type		Deletion		Thapsigargin	
	average	SE	average	SE	average	SE
SERCA *V*_max_, μM/s	1.57	0.02	1.34	0.01	1.26	0.02
InsPR *k*, s^–1^	13.9	0.1	13.3	0.1	11.9	0.3
ER leak *k*, s^–1^	0.108	0.001	0.090	0.001	0.084	0.002
IP3 τ (lifetime), s	34.0	0.2	33.5	0.1	32.1	0.4
IP3 synthesis *k*, μM/s	0.00782	0.00002	0.00796	0.00002	0.00775	0.00006
SERCA *K*_m_, μM	0.0118	0.0001	0.0127	0.0001	0.0123	0.0002

## Conclusion

To date, the described method has enabled detection of the critical differences between the wild-type and transgenic astrocytes in the mouse model of 22q11.2 syndrome in the accompanying article [10]. In the present implementation, both the biophysical characteristics of the employed indicator and the simplification of not considering the finite time of its fluorescence decay in the computational analysis have been methodological choices tuned for the study of intracellular kinetics on a multi-second time scale. Future applications of this method will be able to characterize the intracellular calcium kinetics at a higher temporal resolution using calcium indicators with faster fluorescence decay as well as introducing a decay term in the observation function. Furthermore, the demonstrated [10] use of kinetic inference from time series data supplied by a computer vision algorithm that is capable of handling noisy *in vivo* images establishes an analytical pipeline suitable for future *in vivo* applications. At the same time, given its ability to detect kinetic differences in individual cell cultures, application of this method to patient-derived cells represents a new avenue toward functional molecular diagnostics as part of future individualized medicine approaches.

## Declaration of Competing Interest

The authors declare that they have no known competing financial interests or personal relationships that could have appeared to influence the work reported in this paper.
